# Characterization of a Novel Creeping Tartary Buckwheat (*Fagopyrum tataricum*) Mutant *lazy1*

**DOI:** 10.3389/fpls.2022.815131

**Published:** 2022-04-27

**Authors:** Chenggang Liang, Chunyu Wei, Li Wang, Zhixiu Guan, Taoxiong Shi, Juan Huang, Bin Li, Yang Lu, Hui Liu, Yan Wang

**Affiliations:** ^1^Research Center of Buckwheat Industry Technology, School of Life Sciences, Guizhou Normal University, Guiyang, China; ^2^Guizhou Biotechnology Institute, Guizhou Academy of Agricultural Sciences, Guiyang, China; ^3^Guizhou Key Laboratory of Agricultural Biotechnology, Guiyang, China

**Keywords:** gravitropism, *Pectate lyase 5*, hormone, cellular morphology, transcriptome

## Abstract

Gravity is known as an important environmental factor involved in the regulation of plant architecture. To identify genes related to the gravitropism of Tartary buckwheat, a creeping line was obtained and designated as *lazy1* from the mutant bank by ^60^Co-γ ray radiation. Genetic analysis indicated that the creeping phenotype of *lazy1* was attributed to a single recessive locus. As revealed by the horizontal and inverted suspension tests, *lazy1* was completely lacking in shoot negative gravitropism. The creeping growth of *lazy1* occurred at the early seedling stage, which could not be recovered by exogenous heteroauxin, hormodin, α-rhodofix, or gibberellin. Different from the well-organized and equivalent cell elongation of wild type (WT), *lazy1* exhibited dilated, distorted, and abnormally arranged cells in the bending stem. However, no statistical difference of indole-3-acetic acid (IAA) levels was found between the far- and near-ground bending sides in *lazy1*, which suggests that the asymmetric cell elongation of *lazy1* was not induced by auxin gradient. Whereas, *lazy1* showed up-expressed gibberellin-regulated genes by quantitative real-time PCR (qRT-PCR) as well as significantly higher levels of gibberellin, suggesting that gibberellin might be partly involved in the regulation of creeping growth in *lazy1*. RNA sequencing (RNA-seq) identified a number of differentially expressed genes (DEGs) related to gravitropism at stages I (before bending), II (bending), and III (after bending) between WT and *lazy1*. Venn diagram indicated that only *Pectate lyase 5* was down-expressed at stages I [Log_2_ fold change (Log_2_FC): −3.20], II (Log_2_FC: −4.97), and III (Log_2_FC: −1.23) in *lazy1*, compared with WT. Gene sequencing revealed that a fragment deletion occurred in the coding region of *Pectate lyase 5*, which induced the destruction of a pbH domain in *Pectate lyase 5* of *lazy1*. qRT-PCR indicated that *Pectate lyase 5* was extremely down-expressed in *lazy1* at stage II (0.02-fold of WT). Meanwhile, *lazy1* showed the affected expression of lignin- and cellulose-related genes and cumulatively abnormal levels of pectin, lignin, and cellulose. These results demonstrate the possibility that *Pectate lyase 5* functions as the key gene that could mediate primary cell wall metabolism and get involved in the asymmetric cell elongation regulation of *lazy1*.

## Introduction

Gravity, an important environmental factor, is involved in the regulation of plant architecture. However, the gravitropism of plants is highly complex and related to multistep biological processes, including gravity perception, signal formation in the gravity perceptive cells, intracellular and intercellular signal transduction, and asymmetric cell elongation between the upper and lower sides of the responding organs (Fukaki and Tasaka, 1999; Digby and Firn, 2010). According to the Cholodny-Went theory, the endodermal cells in shoot and columella cells in root have been regarded as the statocytes that mainly perceive gravity (Moritoshi, 1995; Blancaflor and Gilroy, 1998; Fukaki et al., 2002). Besides, statocytes also serve as the site for the formation of biochemical signals (Hensel, 1987). The occurrence of amyloplast sedimentation in statocytes triggers signal transduction and then the asymmetrical distribution of auxin, thus leading to asymmetric cell elongation (Iversen and Larsen, 2010). So far, there has been plenty of evidence collected to support these hypotheses. For example, Arabidopsis *phosphoglucomutase* (*pgm*) mutant exhibits the defect of gravitropism in root and inflorescence caused by the lack of amyloplasts (Toyota et al., 2013). Arabidopsis *shoot gravitropism 2* (*sgr2*), *sgr3*, *sgr4*, *sgr6*, *sgr8*, and *sgr9* exhibit the abnormal gravitropism of inflorescence and hypocotyl induced by aberrant amyloplast sedimentation (Fukaki and Tasaka, 1996; Yoshiro et al., 1997; Kato et al., 2002; Yano et al., 2003; Nakamura et al., 2011; Hashiguchi et al., 2013). However, there is high complexity in the mechanisms for signal transduction and transmission process of gravity signal from amyloplast sedimentation. A variety of secondary messengers, such as Ca^2+^, Insp3, and pH, may be involved in the process of signal transduction and transmission, which transform physical signals into physiological and biochemical signals (Perera, 2006; Urbina et al., 2006; Monshausen et al., 2011). In addition, it has been discovered that the phosphatidylinositol signaling pathway plays a role in regulating gravity signal and auxin polar transport (Mei et al., 2012).

The transport, signaling, and response of auxin have been identified as crucial for plant gravitropism (Salisbury et al., 1988; Zhen et al., 2010; Geisler et al., 2014). In general, there are three families of plant plasma membrane-associated transporters, namely, AUXIN RESISTANT1/LIKE AUX1 (AUX1/LAX) influx carriers, PIN-FORMED (PIN) efflux carriers, and P-GLYCOPROTEIN ATP-binding cassette (PGP) auxin transporters associated with polar auxin transport (Friml, 2003). Arabidopsis *aux1* shows root agravitropic mutation phenotype by reducing auxin uptake within root apex, which indicates the involvement of auxin influx carrier protein AUX1 in root gravitropism regulation (Bennett et al., 1996). Besides, auxin efflux carrier mutants, such as *pin2, pin3*, *pin7*, and *pin8*, exhibit various defects in gravitropism (Bosco et al., 2012; Haga and Sakai, 2013; Rigo et al., 2013). Moreover, there are many modifier proteins, such as GOLVEN secretory peptides and stigmasterol binding protein ROSY1, involved in plant gravitropism by modulating the trafficking dynamics of auxin efflux carriers (Fernandez et al., 2013; Dalal et al., 2016). Arabidopsis DII-VENUS is a sensor involved in the regulation of gravitropism and plant growth by mapping auxin response and distribution (Brunoud et al., 2012).

At present, it remains incompletely understood that the mechanism is for the regulation of asymmetric cell elongation by auxin gradient. A two-phase model for maize stem gravitropism has been proposed through the function research of gravity-stimulated acid invertase gene (*Ivr2*) (Cosgrove, 1999; Long et al., 2002). In general, phase I is regarded as a signaling phase that requires a continuous gravity signal to induce a sufficient indole-3-acetic acid (IAA) gradient. Also, phase II is regarded as the growth phase that involves IAA signal transduction, protein synthesis, *Ivr2* transcripts differentially stimulation, and the differential accumulation of hexose sugars, which leads to differential cell elongation (Cosgrove, 1999; Long et al., 2002). However, this model is unsuited to the explanation of other phenomena, including graviperception and cell wall loosening in maize (Cosgrove, 1999; Long et al., 2002). With experimental and computational approaches taken in combination, a model has been constructed for the apical hook guided by differential growth. The signaling pathways of auxin and ethylene play a role in coordinating both cell division and differential cell growth by mediating various cell division regulatory genes, including *CYCA2;1*, *CYCA2;2*, *CYCA2;3*, *CYCA2;4*, and *SAMBA* (Žádníková et al., 2016).

Crop architecture has a significant impact on yield, such as branching (tiller) number, angle, and plant height, which has long been considered for crop improvement (Coyne, 1980). The plant with prostrate growth phenotype has been first designated as “lazy” in maize mutant due to the defect of gravitropism (Jenkins and Gerhardt, 1931; Van, 1936). Subsequently, there have been many *lazy* mutants discovered in crops, such as rice *oslazy1* (Jones and Adair, 1938) and tomato *lelazy-1* and *lelazy-2* (Roberts, 1984; Hasenstein and Kuznetsov, 1999). Although OsLAZY1 is an uncharacteristic protein with no functional domains, the biochemical function of OsLAZY1 has been proposed to participate in the downstream gravity-sensing mechanism (Abe et al., 1994, 1996, 2010; Li, 2007; Takeshi and Moritoshi, 2007; Akie et al., 2019). The overexpression of *OsPIN2* suppresses *OsLAZY1* expression, thus affecting tiller numbers, angle, and plant height (Chen et al., 2012). In addition, Brevis Radix Like 4 (OsBRXL4), as an interacting protein of OsLAZY1, is also involved in shoot gravitropism and tiller angle regulation by affecting polar auxin transport (Li et al., 2019). As the homolog gene of *OsLAZY1*, maize *ZmLAZY1* can mediate the complex interactions between gravity, auxin, light, and plant growth (Dong et al., 2013; Howard et al., 2014). As a functional ortholog of the *OsLAZY1* gene, *LjLAZY3* plays a vital role in the regulation of root gravitropism (Chen et al., 2019).

Tartary buckwheat [*Fagopyrum tataricum* (L.) Gaertn] is a dicotyledonous plant that belongs to the genus *Fagopyrum* of Polygonaceae. In fact, Tartary buckwheat is usually classified as a cereal that is widely planted in southwest China (Bulan et al., 2017). Modern people prefer to consume Tartary buckwheat for its high nutritional value and health benefits (Zhang et al., 2017; Kalinová et al., 2018; Luo et al., 2020). To gain insight into Tartary buckwheat plant architecture for the research of gene function and high yield breeding, a series of mutation lines were created by ^60^Co-γ ray radiation. Herein, a creeping mutant is reported with impaired shoot gravitropism, designated as *lazy1*. To reveal the mechanisms of *lazy1* for the lack of shoot gravitropism, the phenotypic observation, exogenous hormone treatment, endogenous hormone determination, cell wall structural components measurement, RNA sequencing (RNA-seq), and quantitative real-time PCR (qRT-PCR) analysis were performed. The existing results are expected to provide more insight into the regulation mechanisms for *lazy1* creeping phenotype.

## Materials and Methods

### Mutant Library Creation and Creeping Mutant Screening

There were approximately 30,000 seeds of Tartary buckwheat “cv. Jinqiaomai 2” [wild type (WT)] exposed to ^60^Co-γ ray radiation in the Radiation Center of Guizhou Academy of Agricultural Sciences at 200, 400, and 600 Gy, respectively. All seeds were planted in 2015 during autumn growth season at the Research Center of Buckwheat Industry Technology in Guizhou Province, China (908 m, 26°35′N, 106°52′E) for collection by the individual plant. M_2_ plants were grown in 2016 during spring growth seasons for mutants screening. Subsequently, a creeping mutant, temporarily designated as *lazy1*, was screened through phenotypic observation. For the purpose of genetic stability test, the plants of M_3_, M_4_, and M_5_ were grown in 2016 and 2017 during spring and autumn growth seasons at the Research Center of Buckwheat Industry Technology in Guizhou Province. Besides, the plants of M_6_ were grown in 2018 during winter growth season at the Hainan Breeding and Research Farm of Guizhou Province, China (10 m, 18°45′N, 108°99′E). The plants of M_4_ were backcrossed to WT in 2017, and the progenies of F_2_ were grown in 2018 at the Research Center of Buckwheat Industry Technology in Guizhou Province.

### Pot Experiment

The M_5_ seeds of *lazy1* and WT were planted in 96 cylindrical pots (18.5 cm × 16.2 cm × 13 cm) in 2018, 2019, and 2020 during spring and autumn growth seasons for the purpose of phenotypic and microscopic observation, RNA-sequencing, and metabolites determination. The M_5_ seeds of *lazy1* and WT were planted in 12 rectangle pots (68.5 cm × 38.0 cm × 25.0 cm) in 2018 and 2019 during autumn growth seasons for the investigation into major agronomic traits.

### Gravitropism Test

The plants of *lazy1* and WT at 5 days after germination were horizontally placed, and the stem curvature angle was measured by an angulometer at a 30-min interval for the first 4 h and on a daily basis for the following 2 weeks. The plants were photographed 14 days after treatment. The plants of *lazy1* and WT were inverted by 180° at the time of flowering and photographed 24 h after treatment.

### Exogenous Hormone Treatment

The seeds of *lazy1* and WT were immersed into 50 and 150 mmol/L of heteroauxin, hormodin, α-rhodofix, and gibberellin, respectively. Later, the plants were sprayed with 50 and 150 mmol/L of heteroauxin, hormodin, α-naphthylacetic acid, and gibberellin, respectively, and photographed 3 days after spraying.

### Transmission Electron Microscope Observation

The curved part of *lazy1* stem and the parallel erect part of WT stem at 10 days after germination (bending stage) were sampled and prefixed with a mixed solution of 3% glutaraldehyde. Then, they were postfixed in 1% osmium tetroxide, dehydrated in series acetone, infiltrated in a graded series of acetone solution and propylene oxide, and embedded in Spurr’s epoxy resin. The ultrathin sections were cut with a diamond knife and stained with uranyl acetate and lead citrate. Finally, the microstructure of plant cells was photographed using a Transmission Electron Microscope (JEM-1400PLUS, Tokyo, Japan).

### Endogenous Hormone Determination

The far- and near-ground bending sides of *lazy1* curved stem and the parallel erect parts of WT stem at 10 days after germination were sampled and frozen in liquid nitrogen immediately. Then, the levels of auxin (IAA) and gibberellin (GA_3_) were detected using high-performance liquid chromatography (Rigol L3000, Beijing, China) following the protocol recommended by the manufacturer. The parameters used for IAA measurement are detailed as follows: the ratio at which mobile phase was prepared by mixing methanol and aqueous acetic acid solution is 400:600 ml, the injection volume is 10 μl, the flow rate is 0.8 ml/min, the column temperature is 35°C, the aliasing time is 40 min, the excitation wavelength is 275 nm, and the emission wavelength is 345 nm. The parameters used for GA_3_ measurement are detailed as follows: the ratio at which mobile phase was prepared by mixing methanol and aqueous acetic acid solution is 35:65, the injection volume is 10 μl, the flow rate is 10 ml/min, the column temperature is 30°C, the aliasing time is 30 min, and the detection wavelength is 254 nm.

### Cell Wall Structural Component Contents Measurement

The curved part of *lazy1* stem and the parallel erect part of WT stem at 10 days after germination were sampled for the measurement of major structural components of cell walls. The contents of cellulose, lignin, and pectin were assayed using Cellulose Detection Kit “CLL-1-Y” (Suzhou Comin Biotechnology, Suzhou, China), Lignin Detection Kit “BC4205” (Beijing Solarbio Science & Technology Co., Ltd., Beijing, China), and Pectin Detection Kit “BC1405” in accordance with the instructions from the manufacturer, respectively.

### Agronomic Trait Investigation

A total of 18 plants for each repetition were sampled at harvest for the investigation into branch number, stem node number, and internode length.

### RNA-Seq and Data Analysis

The target stem of *lazy1* and WT at 5 (before bending), 10 (bending), and 15 days (after bending) after germination were sampled and frozen in liquid nitrogen immediately. Total RNAs were extracted using a Plant RNA Kit (Tiangen Biotech, Beijing, China) in the line with the instructions from the manufacturer. The quality of RNAs was examined on 1% agarose gel. RNA concentrations were detected using NanoDrop 2000 spectrophotometer (Thermo Fisher Scientific, Waltham, MA, United States). The sequencing of the library was performed on an Illumina HiSeq platform. The raw data were filtered to trim adaptor sequences while removing low-quality sequences (Q < 20) with >10% uncertain (N) bases. The NCBI database was used to annotate gene function. The criteria of Log_2_ fold change (Log_2_FC) ≥ 1 (or ≤ –1) and false discovery rate (FDR) < 0.01 were applied for the identification of differentially expressed genes (DEGs). DEGs were analyzed using DESeq software. The gene functions of DEGs were annotated by Gene Ontology (GO).^1^ The GO enrichment analysis was conducted using GO:TermFinder software. The Kyoto Encyclopedia of Genes and Genomes (KEGG) database was adopted for DEG pathway enrichment analysis.

### Quantitative Real-Time PCR Analysis

The cDNA was synthesized using the total RNA of the curved stem in *lazy1* and the parallel erect part in WT at 10 days after germination (bending stage) for the detection of qRT-PCR. To explain the phenotype of *lazy1* and verify the reliability of RNA-seq, a total of 25 genes were selected to perform qRT-PCR using SoFast™ EvaGreen^®^ Supermix and CFX96™ (Takara, Dalian, China). Transcript levels were normalized against *Actin.* The cDNA was amplified under the following cycling conditions: (1) one cycle of 95°C for 30 s, (2) 40 cycles of 95°C for 5 s, 60°C for 30 s, and (3) melt-curve from 65 to 95°C by 5 s per step with a 0.5°C increment. The sequences of gene-specific primers are listed in Supplementary Table 1.

### Gene Cloning and Tissue-Specific Expression Analysis of *Pectate lyase 5*

The gene sequence of *Pectate lyase 5* from RNA-seq was applied as the template for PCR analysis. The *Pectate lyase 5* cDNA was amplified with primers F: 5′-ATGAAAAATCTCCATAAATTCTCTCTTC-3′ and R: 5′-TCAGCATTTTTTCCCCTTATTACA-3′. The sequencing of PCR products was performed by Sangong Biotech Co., Ltd. (Shanghai).

The cDNA was synthesized using the total RNA of root, leaf, and flower in WT for the analysis of PCR and gel electrophoresis (2% agarose). The *Pectate lyase 5* cDNA was amplified with primers F: 5′-GATCCAAGACGAGCCATTGT-3′ and R: 5′-ATCACCGTCCGATCTTGTTC-3′ under the following cycling conditions: (1) one cycle of 98°C for 2 min, (2) 35 cycles of 98°C for 10 s, 55°C for 15 s, and 72°C for 1 min, and (3) 72°C for 1 min.

### Statistical Analysis

Data were analyzed using SPSS software (IBM SPSS for Windows, version 19.0, United States). Results are presented as mean ± standard deviation.

## Results

### Phenotypic Characterization of Creeping Mutant *lazy*1

A total of 8,613 lines derived from the mutation bank were screened, and the line “LW600-47-1” was identified as a creeping mutant. Then, 60 seeds of this mutant were sowed, with all of the sprouted plants identified as having the homozygous mutant genotype. Meanwhile, the genetic stability of the creeping phenotype was demonstrated through growth in different seasons (i.e., spring, autumn, and winter) and at various altitudes, latitudes, and longitudes (Guizhou and Hainan). In addition, the creeping and erect plants in the self-progeny of heterozygote plants were segregated at a ratio of 1:3, suggesting that the creeping phenotype of LW600-47-1 was attributed to a single recessive locus (Supplementary Table 2). As the creeping phenotype had been often reported as “lazy” in rice, maize, and tomato, we, therefore, designated the creeping mutant LW600-47-1 as a “lazy” mutant. The “lazy” mutants are often linked with gravity deficiency in rice, maize, and tomato (Jenkins and Gerhardt, 1931; Van, 1936; Jones and Adair, 1938; Roberts, 1984; Hasenstein and Kuznetsov, 1999).

**FIGURE 1:**
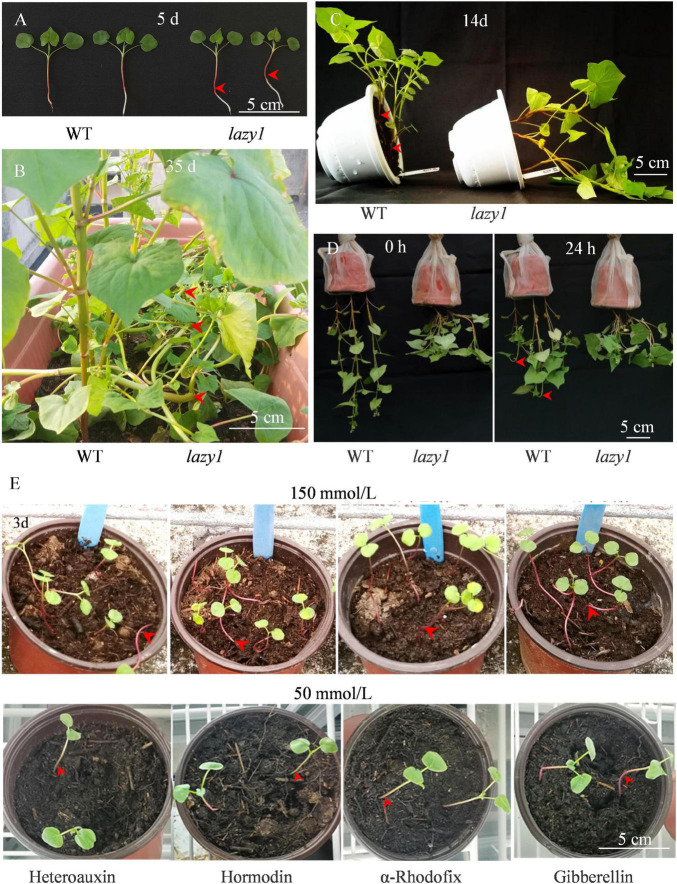
Phenotypic observation of *lazy1* plant at 5 **(A)** and 35 days **(B)** after germination, 14 days after horizontal treatment **(C)**, 0 and 24 h after inverted suspension treatment **(D)**, and 3 days after exogenous hormone treatment **(E)**. Red arrows point to the bending stem. Scale bar = 5 cm.

Compared with WT, the stem of *lazy1* was inflected due to the lack of negative geotropism at the early seedling stage and gradually twisted during growth (Figures 1A,B). At maturity, *lazy1* exhibited a complete creeping phenotype, indicating that the extremely serious defect of gravity response occurred in *lazy1*. Meanwhile, *lazy1* displayed more stem nodes and branches, with branch angle and the length of upper internodes being larger at harvest (Supplementary Figure 1).

**FIGURE 2:**
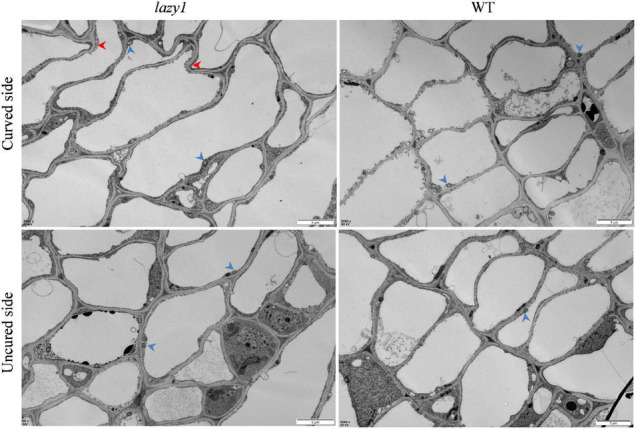
Microscopic observation of *lazy1* bending stem. Red arrows point to the impaired cells wall and blue arrows point to the starch granules. Scale bar = 5 μm.

Different from the well-organized and equivalent cell elongation of WT, the cells of the curved side of *lazy1* were found to be dilated, distorted, and abnormally arranged during observation by transmission electron microscope, which indicates the occurrence of asymmetric cell elongation in *lazy1* bending stem (Figure 2).

### Gravitropism Test and Exogenous Hormone Treatment

To identify the gravity response of *lazy1*, seedlings at 5 days were horizontally placed. A rapid gravity response of WT stem was observed after 1 h of treatment. However, the angle of the *lazy1* stem was unaffected by gravity even after 2 weeks of treatment (Figure 1C and Supplementary Figure 2). Furthermore, an inverted suspension test was performed to confirm the gravity response, and we found rapid negative geotropism in the top organs of WT plants but not *lazy1* mutants after 24-h treatment (Figure 1D). These observations suggested that *lazy1* lacked shoot negative gravitropism.

To determine the effects of the exogenous hormone on the gravity response of *lazy1* mutant, 50 and 150 mmol/L of heteroauxin, hormodin, α-rhodofix, and gibberellin were applied. As shown in Figure 1E, the creeping phenotype of *lazy1* was failed to be recovered by either high or low concentrations of these exogenous hormones.

### Endogenous Hormone Levels and Major Cell Wall Structural Component Contents

**FIGURE 3:**
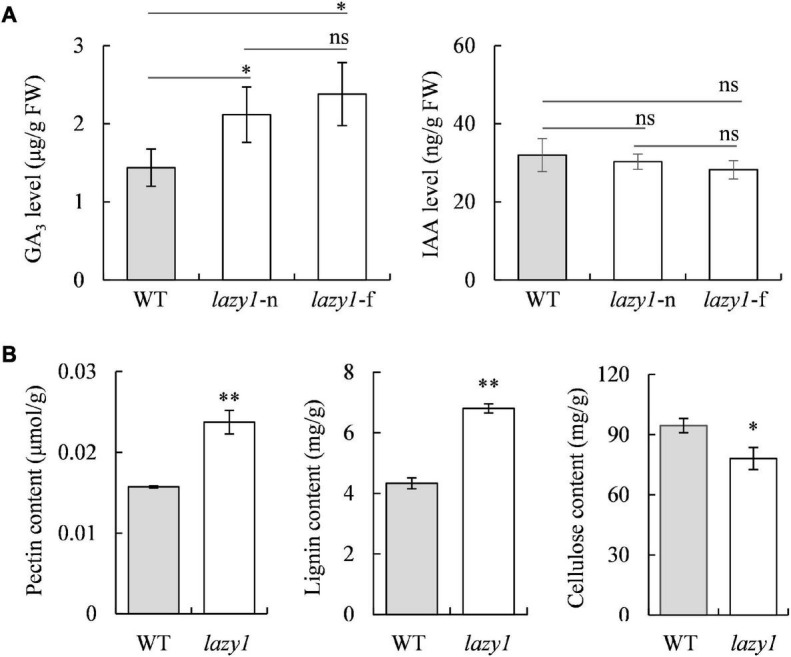
Hormone levels and major cell wall structural components content of *lazy1* bending stem. **(A)** The levels of GA_3_ and IAA in near-ground (*lazy1*-n) and far-ground (*lazy1-*f) side of *lazy1* bending stem. **(B)** The contents of pectin, lignin and cellulose in *lazy1* bending stem. Bars indicate standard deviation (SD, *n* = 4). “ns” indicates no significant difference. “*” and “^**^” indicate the significantly difference at 0.05 and 0.01 levels, respectively.

To determine the effects of endogenous hormone distribution on the creeping growth of *lazy1*, the auxin (IAA) and gibberellin (GA_3_) on the far- and near-ground bending sides were detected. Surprisingly, no statistical difference of IAA levels was found between the far- and near-ground bending sides in *lazy1*, which suggests that the creeping phenotype of *lazy1* was not attributable to the asymmetric distribution of IAA (Figure 3A). Also, the levels of GA_3_ were similar between the far- and near-ground bending sides in *lazy1* (Figure 3A). Compared with WT, *lazy1* exhibited similar levels of IAA but significantly higher levels of GA_3_ on the both far- and near-ground bending sides of *lazy1* (Figure 3A). Moreover, the contents of major cell wall structural materials in the *lazy1* bending stem were detected. Compared with WT, *lazy1* showed significantly higher contents of pectin and lignin but significantly lower content of cellulose (Figure 3B), indicating a disruption caused to the metabolism related to cell wall structure in *lazy1*.

### RNA-Seq and Gene Ontology Analysis

**FIGURE 4:**
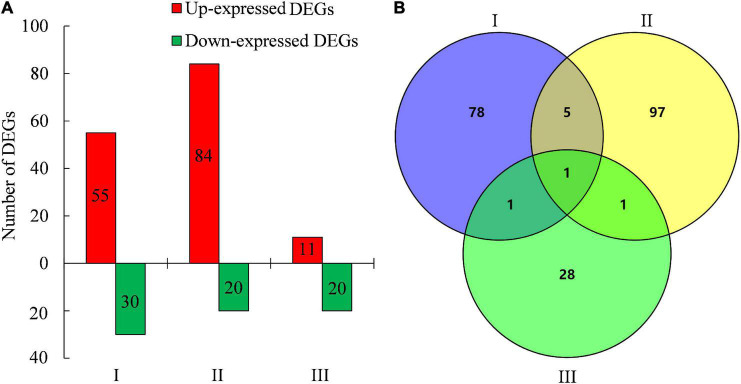
Analysis of DEGs and Venn between WT and *lazy1* at stage I (before bending), II (bending), and III (after bending) by RNA-seq. **(A)** The number of up-expressed and down- expressed DEGs between WT and *lazy1***. (B)** The number of common DEGs between WT and *lazy1* by Venn analysis.

To gain insights into the molecular mechanisms of creeping phenotype in *lazy1*, the RNA-seq of the stem was performed at stages I (before bending), II (bending), and III (after bending), respectively. Based on the threshold values of Log_2_ ratio ≥ 1 and FDR < 0.05, a total of 85, 104, and 31 genes were identified as DEGs at stages I, II, and III, respectively, between *lazy1* and WT, out of which 55, 84, and 11 DEGs were found up-expressed and 30, 20, and 20 DEGs were shown to be down-expressed in *lazy1*, respectively (Figure 4A, Supplementary Figure 3, and Supplementary Table 3). The Venn diagram indicated one common DEG among stages I, II, and III, six common DEGs between stages I and II, two common DEGs between stages I and III, and two common DEGs between stages I and III (Figure 4B).

**FIGURE 5:**
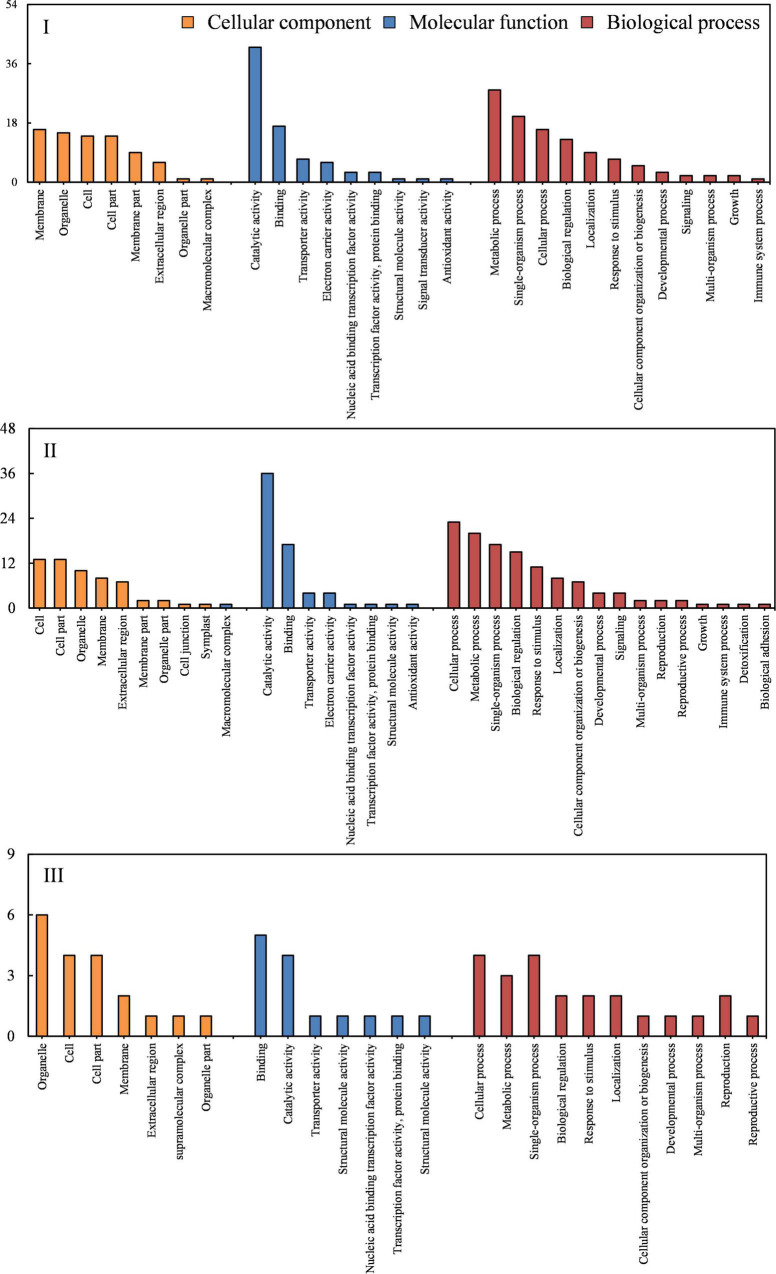
Gene ontology (GO) analysis of DEGs at stage I (before bending), II (bending), and III (after bending).

**FIGURE 6:**
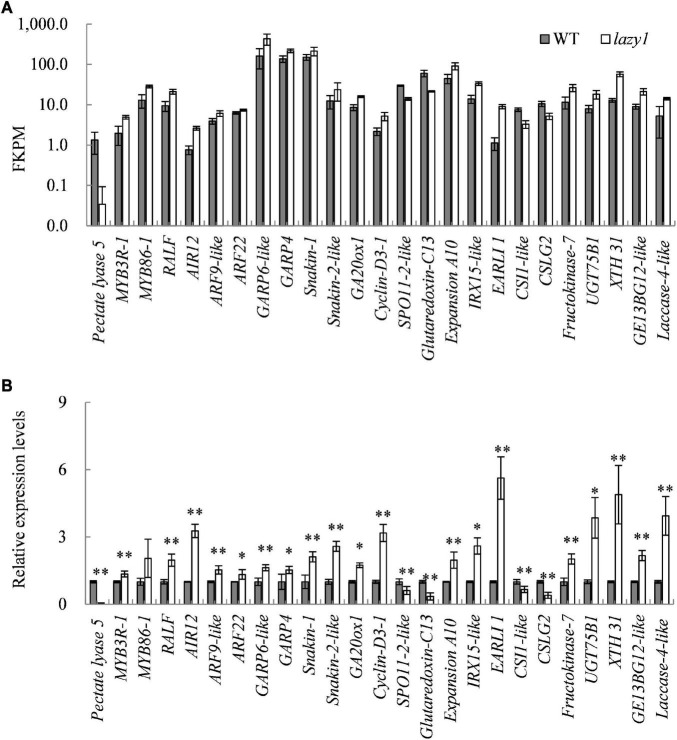
Quantitative real-time PCR analysis of DEGs related to *lazy1* phenotype. **(A)** The FKPM value of genes by RNA-seq. **(B)** The relative expression levels of gene by qRT-PCR. Bars indicate standard deviation (SD, *n* = 4). * and ^**^ indicate the significant difference at 0.05 and 0.01 levels, respectively between WT and *lazy1.*

The GO analysis was further performed to identify the significantly enriched categories of genes (Figure 5). The “metabolic process,” “cellular process,” and “single-organism process” were identified as the most dominant GO terms enriched in the biological process category; the “cell,” “cell part,” and “organelle and membrane” were determined as dominant in the cellular component category; the “catalytic activity” and “binding” were revealed to be dominant in the molecular function category (Figure 6).

### Analysis of Common Differentially Expressed Genes

**FIGURE 7:**
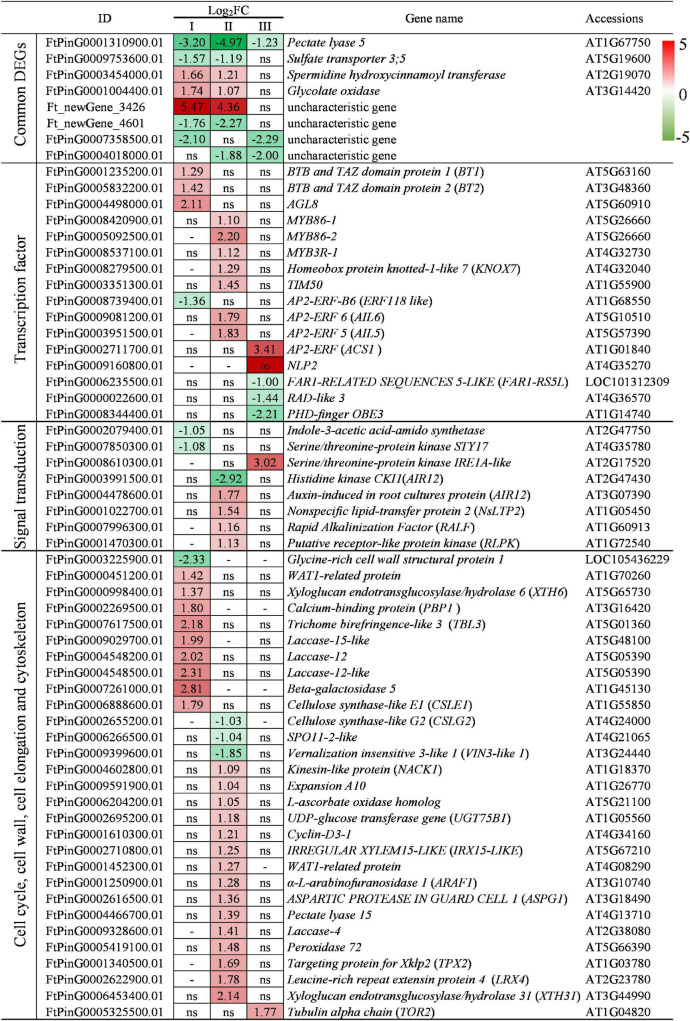
Analysis of DEGs at stage I (before bending), II (bending), and III (after bending). “ns” indicates no significant difference of gene expression was detected between WT and *lazy1.* “-” indicates the gene expression level was not detected.

Compared with WT, *lazy1* has 5 down-expressed and 3 up-expressed common DEGs that are altered in two or three examined stages (Figure 7). Among them, only *Pectate lyase 5*, which is capable to catalyze the dissolution of pectin chains and regulate primary cell walls (Palusa et al., 2007), was found to be down-expressed at stages I, II, and III in *lazy1* stem. *Sulfate transporter 3;5*, a low-affinity sulfate transporter, was found to be down-expressed at stages I and II in *lazy1* stem (Kataoka et al., 2004). In contrast, there were another two up-expressed common DEGs, namely, *spermidine hydroxycinnamoyl transferase* and *glycolate oxidase*. In addition, some common DEGs have not yet been functionally characterized, including *Ft_newGene_4601*, *FtPinG0007358500.01*, *FtPinG0004018000.01*, and *Ft_newGene_3426* (Figure 7).

### Analysis of Transcription Factor

Compared with WT, *lazy1* has 10 up-expressed and four down-expressed DEGs belonging to the transcription factor families, including MADS-box, BTB and TAZ, MYB, AP2/ERF, KNOX, NLI AP2/ERF, NLP FAR1, PHD-finger, and RAD (Figure 7). Among them, *BT1* and *BT2* were found to be up-expressed in *lazy1* at stage I, which positively regulated the auxin signaling in the initial response to the gravity of *lazy1* (Mandadi et al., 2009; Robert et al., 2009). Meanwhile, *TIM50* and *KNOX7* were found to be up-expressed in *lazy1* at stage II, which positively regulated the cell wall biosynthesis and asymmetry cell elongation of *lazy1*, respectively (Kumar et al., 2012; Wang et al., 2020). Furthermore, *RAD-like 3* was found to be down-expressed in *lazy1* at stage III, which negatively regulated the asymmetry cell elongation of *lazy1* (Baxter et al., 2007). In addition, many genes related to plant meristems development and organ growth were found to be up-expressed in *lazy1* at stages I, II, and III, respectively, including *MYB3R1*, *MYB86* (Kobayashi et al., 2015), *AIL5*, *AIL6* (Ouakfaoui et al., 2010), *ACS1* (Rodrigues-Pousada et al., 1993), and *AGL8* homolog (Mandel and Yanofsky, 1995), while the shoot meristem-related PHD-finger gene *OBE3* (Ta-Fang et al., 2016), transposase-derived transcription factor gene *FAR1-RS5L* (Lin and Li, 2018), and *ERF118*-*like* (Seyfferth et al., 2018) were found to be down-expressed in *lazy1*.

### Analysis of Differentially Expressed Genes in Signal Transduction

Compared with WT, *lazy1* has five up-expressed and two down-expressed DEGs involved in signal transduction (Figure 7). Among them, *Indole-3-acetic acid-amido synthetase* was found to be down-expressed in *lazy1* at stage I, and *AIR12* was found to be up-expressed in *lazy1* at stage II, suggesting that the metabolism and signaling of auxin were affected in *lazy1* (Zhang et al., 2009; Gibson and Todd, 2015). Meanwhile, the genes related to plant growth and development were found to be up-expressed in *lazy1* at stages II and III, respectively, including *NsLTP2* (Thoma et al., 1994), *RLPK* (Kanamoto et al., 2002), *RALF* (Liam and Turner, 2017), and *IRE1A-like* (Bao et al., 2019). The chloroplast differentiation-related gene *STY17* (Eisa et al., 2019) and cytokinin-related gene *CKI1* (Dobisova et al., 2017) were found to be down-expressed in *lazy1* at stages I and II, respectively.

### Analysis of Differentially Expressed Genes Involving in Cell Cycle, Cell Wall, Cell Elongation, and Cytoskeleton

Compared with WT, *lazy1* has plenty of DEGs involved in cell cycle, cell wall, cell elongation, and cytoskeleton (Figure 7). Among them, *Glycine-rich cell wall structural protein 1* was found to be down-expressed in *lazy1* at stage I, while *Expansion A10* (Hur et al., 2014), *WAT1-related protein gene* (Ranocha et al., 2010; Grones and Friml, 2015), *ARAF1* (Chavez Montes et al., 2008), and *LRX4* (Herger et al., 2020) were found to be up-expressed in *lazy1* at stages I and II, respectively, suggesting that the regulation of cell wall structure was affected in *lazy1*. The abnormal expression of genes related to the metabolism of pectin, lignin, and cellulose was identified in *lazy1* at stages I, II, and III, respectively, including *Pectate lyase 5* (Palusa et al., 2007), *Pectate lyase 15* (Palusa et al., 2007), *PBP1* (Benjamins, 2003), *TBL3* (Bischoff et al., 2010), *Laccase-12*, *Laccase-12-like*, *Laccase-15-like*, *Laccase-4* (Liu et al., 1994; Berthet et al., 2011), *beta-galactosidase 5* (Gantulga et al., 2008), *IRX15-LIKE* (Brown et al., 2011), *XTH6*, *XTH31* (Ryusuke and Kazuhiko, 2001), *CSLE1* (Liepman et al., 2005), and *CSLG2* (Xu et al., 2020). Meanwhile, *VIN3-like 1* was found to be down-expressed in *lazy1* at stage II, which regulated the branching angle of *lazy1* (Zhao et al., 2010). *UGT75B1* was found to be up-expressed in *lazy1* at stage II, which modulated the auxin-ethylene cross-talk for gravitropism (Nziengui et al., 2018). *TOR2* was found to be up-expressed in *lazy1* at stage III, which regulated the twisting growth of *lazy1* (Buschmann et al., 2009). In addition, some genes related to cell cycle and redox were found to be abnormally expressed in *lazy1* at stages I and II, respectively, including *TPX2* (Beáta et al., 2013), *Cyclin-D3-1* (Bartkova et al., 1998; Randall et al., 2015), *NACK1* (Tanaka et al., 2004), *L-ascorbate oxidase homolog* (Lim, 2012), and *Peroxidase 72* (Fernández-Pérez et al., 2015).

### Analysis of Genes Probably Involving in Creeping Growth Regulation of *lazy*1

To identify the genes that are probably related to the creeping growth of *lazy1* mutant, a total of 25 genes, which are responsible for regulating signal transduction, cell cycle, cell wall, and cell elongation, were selected for qRT-PCR detection at the bending stage of *lazy1*. As expected, the expression of all selected genes between *lazy1* and WT showed a trend that is consistent with the transcriptome data (Figure 6). Compared with WT, the auxin response factor genes *AIR12, ARF9-like*, and *ARF22* were up-expressed in *lazy1* stem, suggesting that the auxin response was enhanced in *lazy1*. In addition, the gibberellin-regulated protein genes, including *GA20ox1*, *GRP4*, *GRP6-like*, *Snakin-1*, and *Snakin-2-like*, were up-expressed, implying that both GA biosynthesis and response were enhanced in *lazy1*. Furthermore, the cell cycle-related gene *Cyclin-D3-1* was up-expressed, but *SPO11-2-like* was down-expressed. Besides, the cellulose-related genes *CSLG2* and *CSI1-like* were down-expressed, while the xyloglucan- and lignin-related genes *GE13BG12-like*, *XTH 31*, *UGT75B1*, *IRX15-like*, *Laccase 4*, and *Fructokinase 7* were up-expressed in *lazy1* stem. Notably, *Pectate lyase 5* was extremely down-expressed in *lazy1* stem, the level of which was only one fifties of WT.

### Gene Cloning and Tissue-Specific Expression of *Pectate lyase 5*

**FIGURE 8:**
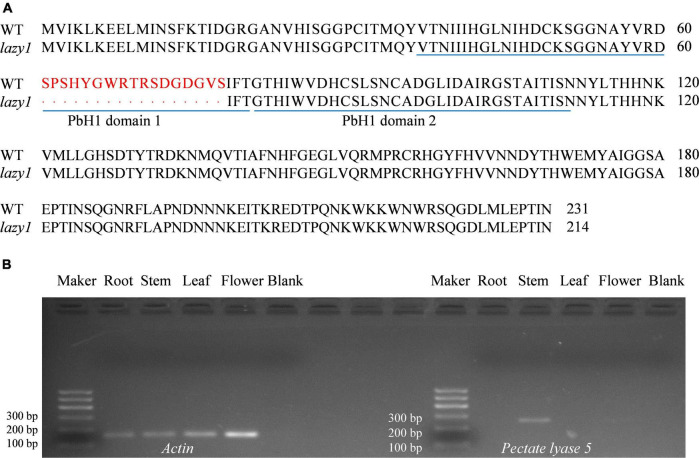
Alignment of *Pectate lyase 5* amino acid sequence **(A)** and the tissue specific expression of *Pectate lyase 5* **(B)**. Red letters indicate the different amino acid sequences between WT and *lazy1.* Blue lines are the locations of pbH domains.

To analyze the sequence of *Pectate lyase 5* between *lazy1* and WT, PCR-based cloning was performed. *Pectate lyase 5* encodes a 231-amino acid protein containing two pbH domains. According to nucleotide sequencing, a fragment deletion was detected in the coding region of *Pectate lyase 5* in *lazy1*, which induced the destruction of pbH domain 1 and the changes of three-dimensional structure in *Pectate lyase 5*, as compared to WT (Figure 8A and Supplementary Figures 4, 5).

Meanwhile, the PCR-based gene expression of *Pectate lyase 5* was performed using WT as material. According to the analysis of gene tissue-specific expression, *Pectate lyase 5* was identified to be rarely expressed in root, leaf, and flower but highly expressed in stem (Figure 8B).

## Discussion

### Tartary Buckwheat *lazy*1 Was a Creeping Mutant Lacking Shoot Negative Gravitropism

The negative geotropic growth of crop shoot is crucial for plant architecture, colony growth, and yield formation (Coyne, 1980). At present, prostrate or creeping phenotype has been reported as a “lazy” growth habit in various plants, such as rice, tomato, maize, barley, and *Arabidopsis* (Jones and Adair, 1938; Roberts, 1984; Türkan and Suge, 1991; Howard et al., 2014; Taniguchi et al., 2017). Herein, a novel Tartary buckwheat mutant (*lazy1*) with a “lazy” growth habit was reported by means of ^60^Co-γ ray radiation. Compared with WT, *lazy1* displayed curving stem at the early seedling stage and fully prostrated during growth due to the extremely serious defect of shoot gravitropism. In addition, *lazy1* exhibited impervious growth during both horizontal and inverted suspension tests, which indicates that *lazy1* was completely lacking in shoot negative gravitropism.

### Tartary Buckwheat *lazy*1 Displayed Aberrant Cell Structure With Normal Auxin Distribution

It is widely known that hormones are responsible for the regulation of plant gravitropism (Venbrinkab and Kiss, 2019; Yin et al., 2019). According to the Cholodny-Went theory, the asymmetric distribution of auxin plays a crucial role in the regulation of plant gravitropism (Zhen et al., 2010; Venbrinkab and Kiss, 2019). Auxin has been demonstrated as the major hormone that is effective not only in triggering lignin deposition and cell wall strengthening in the far-ground bending side (Naoki and Yoshio, 1978) but also in inducing pectate lyase to remodel the cell wall during the course of cell elongation and differentiation (Domingo et al., 1998). Lignin, cellulose, and pectin are known as the major cell wall structural components in the shoot of plants. In this study, it was discovered that the contents of lignin, cellulose, and pectin in the bending stem of *lazy1* were significantly affected, compared with WT (Figure 3). However, there was no asymmetric distribution of auxin detected on both far- and near-ground bending sides in *lazy1*. Despite this, *lazy1* did show dilated, distorted, and abnormally arranged cells in the bending stem. Therefore, it was speculated that the creeping phenotype of *lazy1* was regulated by the abnormal metabolism of cell wall structural components.

Despite insufficient evidence related to the regulation of gravitropism by gibberellins, the role of gibberellins in the differential shoot growth following gravitropism has been demonstrated (Rood et al., 1987). Probably, gibberellins could act as the modulator of auxin sensitivity, which reduces the response to gravity but increases the variance of this process (Rodrigo et al., 2011). In this study, *lazy1* exhibited not only significantly higher levels of GA_3_ but also a larger length of upper internodes, compared to WT. Meanwhile, qRT-PCR revealed that the gibberellin-regulated genes were up-expressed in the bending stem of *lazy1* (Figure 6B), which suggests the possible involvement of gibberellins in the process of shoot gravitropism.

### Transcriptome Analysis Suggests Mechanisms for Lacking Shoot Gravitropism

As a directional response to gravity stimulus, plant gravitropism is regulated by complex signaling and metabolic networks (Venbrinkab and Kiss, 2019). The secondary messenger Ca^2+^ is suggested to be involved in the transduction and transmission of the signal after the gravity signal (Monshausen et al., 2011). Calcium and BTB proteins play their specific roles in auxin transport (Mandadi et al., 2009; Robert et al., 2009). In this study, the calcium-binding protein gene *PBP1* and BTB protein genes *BT1* and *BT2* were found to be up-expressed before bending in *lazy1* (Figure 7). Despite no detection of DEGs related to auxin transport between *lazy1* and WT, the auxin-related gene *Indole-3-acetic acid-amido synthetase* was down-expressed in *lazy1* before bending. Rice *tled1-D* mutant shows an increase in both leaf angle and tiller numbers, which is attributed to the defective function of *Indole-3-acetic acid-amido synthetase* (Zhang et al., 2009). Therefore, it is inappropriate to completely rule out the roles of auxin signaling in the initial response to gravity in *lazy1* before bending.

**FIGURE 9:**
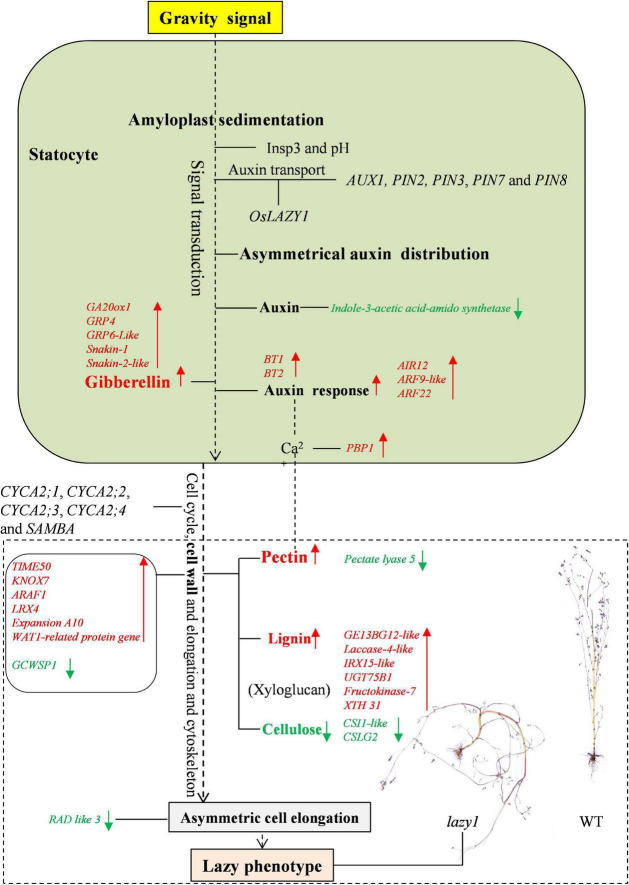
The model for the regulation of shoot gravitropism in *lazy1* based on RNA-seq and qRT-PCR. Red arrows represent the elevated contents of metabolite or up-expressed levels of gene in *lazy1*. Green arrows represent the decreased contents of metabolite or down-expressed levels of gene in *lazy1.*

In addition to the Cholodny-Went theory, some other new hypotheses have been put forward in relation to the principal mechanism of gravitropic growth regulation. Not only does gravistimulation inhibit the infiltration of auxin-induced wall-loosening factors into the extension-restricting epidermal walls, but it also leads to differential growth temporarily (Edelmann, 2010). It is suggested that plant gravitropism is subjected to regulation by the different sensitivity between the upper and lower sides under gravity stimulus (Evans, 1992). The lateral distribution of gravity-induced growth inhibitors is suspected to inhibit auxin activity in the upper side organ (Tokiwa et al., 2006). Based on RNA-seq and qRT-PCR, the signaling and metabolic networks of asymmetric cell elongation regulation for *lazy1* are illustrated in Figure 9.

It was common for the asymmetric cell elongation to occur in the mutants lacking in gravitropism. Plant cell elongation is determined by not only turgor pressure but also mechanical characteristics of the cell wall (Taiz, 1984). In this study, *lazy1* showed a large number of DEGs related to cell division, cell wall, cell elongation, and cytoskeleton (Figure 7), as well as the abnormal cell structure on the bending side (Figure 2). Particularly, the abnormal expression of *Glycine-rich cell wall structural protein 1*, *Expansion A10*, *WAT1-related protein gene*, *ARAF1*, and *LRX4* was detected in *lazy1* at stages I and II, respectively, suggesting that the regulation of cell wall structure was affected in *lazy1* (Ryser et al., 1997; Chavez Montes et al., 2008; Ranocha et al., 2010; Hur et al., 2014; Grones and Friml, 2015; Herger et al., 2020). Cell-wall polysaccharides play an important role in the control of the extensibility of the cell wall (Taiz, 1984). The cell wall metabolism is closely associated with gravity response (Ikushima et al., 2008). The degradation of cell wall xyloglucan accelerates stem elongation and blocks upright-stem gravitropism in poplar through the overexpression of *xyloglucanase* (Hayashi and Kaida, 2011). Compared to WT, *lazy1* exhibited up-expressed xyloglucan endotransglucosylase/hydrolases gene *XTH6* and xyloglucan acetylation gene *TBL3* before bending and also *IRX15-like* and *XTH31* at the bending stage (Figure 7). Cellulose is involved in the hypocotyl graviresponses regulated by brassinosteroids in *Arabidopsis* (Marc et al., 2021). Lignin plays a vital role in the generation of maturation stress for normal and compression wood by increasing the compressive stress applied to the cell wall matrix (Okuyama et al., 1998). The viscosity of pectin is negatively correlated with the growth rate in mung bean hypocotyl (Taiz, 1984). There were many DEGs related to the metabolism of lignin, pectin, and cellulose, as identified between *lazy1* and WT (Figure 7), which is consistent with the results of the change to lignin, pectin, and cellulose contents in *lazy1* bending stem (Figure 3). Therefore, it is suggested that the disrupted cell wall metabolism induces the occurrence of asymmetric cell elongation and leads to the defect in the ability of plants to withstand gravity, which enhances the creeping growth of *lazy1*.

### Tartary Buckwheat *lazy*1 Showed Genetic Mutation of *Pectate lyase 5*

*LAZYs* are involved in gravitropism regulation when environmental signals are converted into differential growth, both after amyloplast sedimentation (Abe et al., 1994) and before auxin gradient formation (Godbolé et al., 1999; Li, 2007; Takeshi and Moritoshi, 2007). Among various *LAZYs*, only *LAZY1* was reported to regulate shoot gravitropism in rice (Li, 2007; Takeshi and Moritoshi, 2007), maize (Dong et al., 2013), and *Arabidopsis* (Taniguchi et al., 2017). Rice *OsLAZY1* functions as a negative regulator of polar auxin transport (Li, 2007). However, it is possible for the target gene of Tartary buckwheat *lazy1* not to be the homologous gene of *OsLAZY1*, because the indistinguishable levels of auxin were detected between WT and *lazy1*.

A time-series gene expression data were screened based on RNA-seq (Figure 7 and Supplementary Table 3). Compared with WT, only *Pectate lyase 5* down-expressed at stages I, II, and III in *lazy1*. That is, the metabolism of pectin in *lazy1* was affected throughout the gravity response. Pectin molecules can be used to build cross-links through calcium involved in the regulation of cell wall strength and growth for expanding organs (Yamaoka and Chiba, 1983; Knox et al., 1990; Peaucelle et al., 2011). Pectate lyase catalyzes the dissolution of pectin chains for the loosening, remodeling, and rearrangement of the cell wall (Baker et al., 1990; Palusa et al., 2007). *Pectate lyase* genes play important and diverse roles in the development of different plant tissues (Palusa et al., 2007; Wieczorek et al., 2014; Sun et al., 2018; Yang et al., 2020). Most *Pectate lyase* genes are preferentially expressed in flowers of the plants (Palusa et al., 2007; Sun et al., 2018), while Tartary buckwheat *Pectate lyase 5* exhibited tissue-specific expression in stem (Figure 8B). Meanwhile, qRT-PCR revealed that *Pectate lyase 5* was rarely expressed at stage II (0.02-fold of WT) in the bending stem of *lazy1* (Figure 6B). As expected, Tartary buckwheat *lazy1* harbors genetic mutation of *Pectate lyase 5* according to gene sequencing (Figure 8A and Supplementary Figure 4). Rice *rbh1-1* mutant shows twisty and whitish spikelet due to the defective function of pectate lyase (He et al., 2021). In this study, Tartary buckwheat *lazy1* exhibited twisty and groveling stem with increased pectin content. The expression of *Pectate lyase* has been confirmed to be significantly up-regulated by auxin induction (Domingo et al., 1998). The pectin-related genes, including *Pectin lyase-like*, *Pectin lyase 2*, *Pectinesterase-like*, and *Pectin-glucuronyltransferase*, are differentially expressed between compression wood and opposite wood (Li et al., 2013). Therefore, it is speculated that the genetic mutation of *Pectate lyase 5* could induce the defect function of pectate lyase and disrupt cell wall metabolism, which leads to asymmetric cell elongation growth and affects the shoot gravitropism of *lazy1*.

## Data Availability Statement

The original contributions presented in the study are publicly available. This data can be found here: National Center for Biotechnology Information (NCBI) BioProject database under accession number, PRJNA007201.

## Author Contributions

YW conceived the original research plan and designed the experiment. CL, CW, and LW wrote the manuscript, whereas YW reviewed and edited the manuscript. All authors performed the experiments, analyzed the data, and approved the submitted version.

## Conflict of Interest

The authors declare that the research was conducted in the absence of any commercial or financial relationships that could be construed as a potential conflict of interest.

## Publisher’s Note

All claims expressed in this article are solely those of the authors and do not necessarily represent those of their affiliated organizations, or those of the publisher, the editors and the reviewers. Any product that may be evaluated in this article, or claim that may be made by its manufacturer, is not guaranteed or endorsed by the publisher.

## Footnotes

1
http://www.geneontology.org/
