# Supplementary material for: Characterization of a Novel Creeping Tartary Buckwheat (Fagopyrum tataricum) Mutant lazy1
Source: Front Plant Sci. 2022 Apr 27;13:815131. doi: 10.3389/fpls.2022.815131 (PMC9094088; doi:10.3389/fpls.2022.815131)
Supplement: Supplementary file 1 [file Table_1.DOCX]

**Supplementary Table 1** The primer sequence of genes for qRT-PCR

|  | ID | Gene |  | Primers |
| --- | --- | --- | --- | --- |
| 1 | FtPinG0008597200.01 | *Auxin response factor 9-like*  (*ARF9-like*) | F | ATTGGTTTCGGGTGATTCTGTC |
|  |  |  | R | CTCGGCGGTAACTAAGTCTGCT |
| 2 | FtPinG0002645500.01 | *Auxin response factor 22*  (*ARF22*) | F | ATTCTCCGTTCCTCGTTACTGC |
|  |  |  | R | CTCCAGACAACGCCGTGAAT |
| 3 | FtPinG0006944700.01 | *Gibberellin-regulated protein 4*  (*GRP4*) | F | GAGTGTGCCCCAAACTCAAT |
|  |  |  | R | ACCCCTCTTGGTCTTCCAGT |
| 4 | FtPinG0006350100.01 | *Gibberellin -regulated protein 6-like* (*GRP6-like*) | F | TTCTCCTCGTCGCCTTTCTA |
|  |  |  | R | AACATGCATGGCTTGTGGTA |
| 5 | FtPinG0001037700.01 | *Gibberellin -regulated protein Snakin-1* | F | GAAGGCCTTGTATGCAGCTC |
|  |  |  | R | CTGCCTTGGCACACCTATTT |
| 6 | FtPinG0002727000.01 | *Gibberellin -regulated protein Snakin-2-like* | F | ACCAGTATCCGCTCCAACTG |
|  |  |  | R | CAGTTGCAAGTGTGACAGCA |
| 7 | FtPinG0008198800.01 | *Gibberellin 20* *oxidase 1*  (*GA20ox1*) | F | ATCCCACCTCCCTTACCATC |
|  |  |  | R | CCTATTCACCACTGCCCTGT |
| 8 | FtPinG0001310900.01 | *Pectate lyase 5* | F | GATCCAAGACGAGCCATTGT |
|  |  |  | R | ATCACCGTCCGATCTTGTTC |
| 9 | FtPinG0006591600.01 | *Cellulose synthase interactive 1-like* (*CSI1-like*) | F | TCACCCTAGCCAAAAGCAAC |
|  |  |  | R | TAATCTCCGTCACCCTTCCA |
| 10 | FtPinG0002655200.01 | *Cellulose synthase-like G2*  (*CSLG2*) | F | GCGTTGCAGCCTTTCTATTC |
|  |  |  | R | AGGAAACAAGCCAGGAAGGT |
| 11 | FtPinG0009591900.01 | *Expansion A10*  (*EXP A10*) | F | GAACAATGGGTGGAGCTTGT |
|  |  |  | R | TACACCAGCCACCTGCATTA |
| 12 | FtPinG0009372700.01 | *Fructokinase-7* | F | TTAGTTGGACGCCTGGAACC |
|  |  |  | R | ACCCTGACAACGTGAAGCAA |
| 13 | FtPinG0002695200.01 | *Xylan alpha-glucuronosyltransferase 1-like* (*XAGLT1-like*) | F | CCTTTTCCGGCCAAAACGAG |
|  |  |  | R | ACTCGGCCTAGTGATGGAGT |
| 14 | FtPinG0001610300.01 | *Cyclin-D3-1* | F | TTGACAGCCGTTCTCTCCAC |
|  |  |  | R | CGAGTCGCCGGATTATGTGA |
| 15 | FtPinG0002665200.01 | *glucan Endo-1,3-beta-glucosidase 12-like* (*GE13BG12-lke*) | F | GTTCAAACTCTCCGCCTCCA |
|  |  |  | R | GGGGTTGGTGCTGGAATACA |
| 16 | FtPinG0002710800.01 | *IRX15-like* | F | AAACAGGGGAGCTCGAACAG |
|  |  |  | R | CCGGTCGGGTTAGTGATGAG |
| 17 | FtPinG0009328600.01 | *Laccase-4-like* | F | CGAGCAAGCCAATCATCACG |
|  |  |  | R | CGTCTGACCAACAACTCCGA |
| 18 | FtPinG0006453400.01 | *Xyloglucan endotransglucosylase/hydrolase protein 31* (*XETG/H 31*) | F | TGGCAAGTACAAGGCGGATT |
|  |  |  | R | ATTCTGGGGTAAGTGCACGG |
| 19 | FtPinG0008420900.01 | *MYB86-1* | F | GAGAGGCTTGTGGTCTCCTG |
|  |  |  | R | GGCCCATCTGTTTCCTACAA |
| 20 | FtPinG0008537100.01 | *MYB3R-1* | F | CCTTCACATGCACACCCTCT |
|  |  |  | R | GTTTGTGGCGGGTCAACTTC |
| 21 | FtPinG0005701300.01 | *Glutaredoxin-C13* | F | GGGTTACAACCCAAACATGC |
|  |  |  | R | CAAAGGCTGGTAAGGCTTGA |
| 22 | FtPinG0006266500.01 | *SPO11-2-like* | F | CAAGCGAGCTGACATTGAAG |
|  |  |  | R | GCTACTTTGCCGTGTCCATT |
| 23 | FtPinG0007996300.01 | *Rapid Alkalinization Factor* (*RALF*) | F | AGCTTGCGATGGATCTCTGG |
|  |  |  | R | ACTGCAACTCCGGCTATACG |
| 24 | FtPinG0004478600.01 | *Auxin-induced in root cultures protein* (*AIR12*) | F | TGGAACCCGGAAAGAAGTGG |
|  |  |  | R | CCTCCTGAGAACCACAACCC |
| 25 | FtPinG0004021800.01 | *Lipid transfer protein* (*EARLI 1*) | F | ACCTTGAAACTCGGGGTGTG |
|  |  |  | R | GGTACAAAGACAAACCGCGG |
| 26 |  | *Actin* | F | GAAATTCGCAAGTACCAGAAGAG |
|  |  |  | R | CCAACAAGGTATGCCTCAGC |
