# Supplementary material for: Characterization of a Novel Creeping Tartary Buckwheat (Fagopyrum tataricum) Mutant lazy1
Source: Front Plant Sci. 2022 Apr 27;13:815131. doi: 10.3389/fpls.2022.815131 (PMC9094088; doi:10.3389/fpls.2022.815131)
Supplement: Supplementary file 2 [file Table_2.DOCX]

**Supplementary Table 2** Segregation of creeping (C) and erect (E) plants in the selfprogeny of heterozygote plants by *lazy1* × WT

| Combination | Total plants | Creeping plants | Erect plants | Expected ratio (C:E) | χ^2^ |
| --- | --- | --- | --- | --- | --- |
| F_2_ | 33 | 8 | 27 | 1:3 | 0.21 |
| F_2_ | 26 | 5 | 21 | 1:3 | 0.46 |
| F_2_ | 42 | 9 | 33 | 1:3 | 0.29 |
| F_2_ | 51 | 12 | 39 | 1:3 | 0.06 |
| F_2_ | 46 | 10 | 36 | 1:3 | 0.26 |
| F_2_ | 68 | 16 | 52 | 1:3 | 0.08 |

Note: χ^2^ < 3.84, *P* > 0.05; χ2 < 6.63, *P* > 0.01.
