# Supplementary figures and images for: Characterization of a Novel Creeping Tartary Buckwheat (Fagopyrum tataricum) Mutant lazy1
Source: Front Plant Sci. 2022 Apr 27;13:815131. doi: 10.3389/fpls.2022.815131 (PMC9094088; doi:10.3389/fpls.2022.815131)

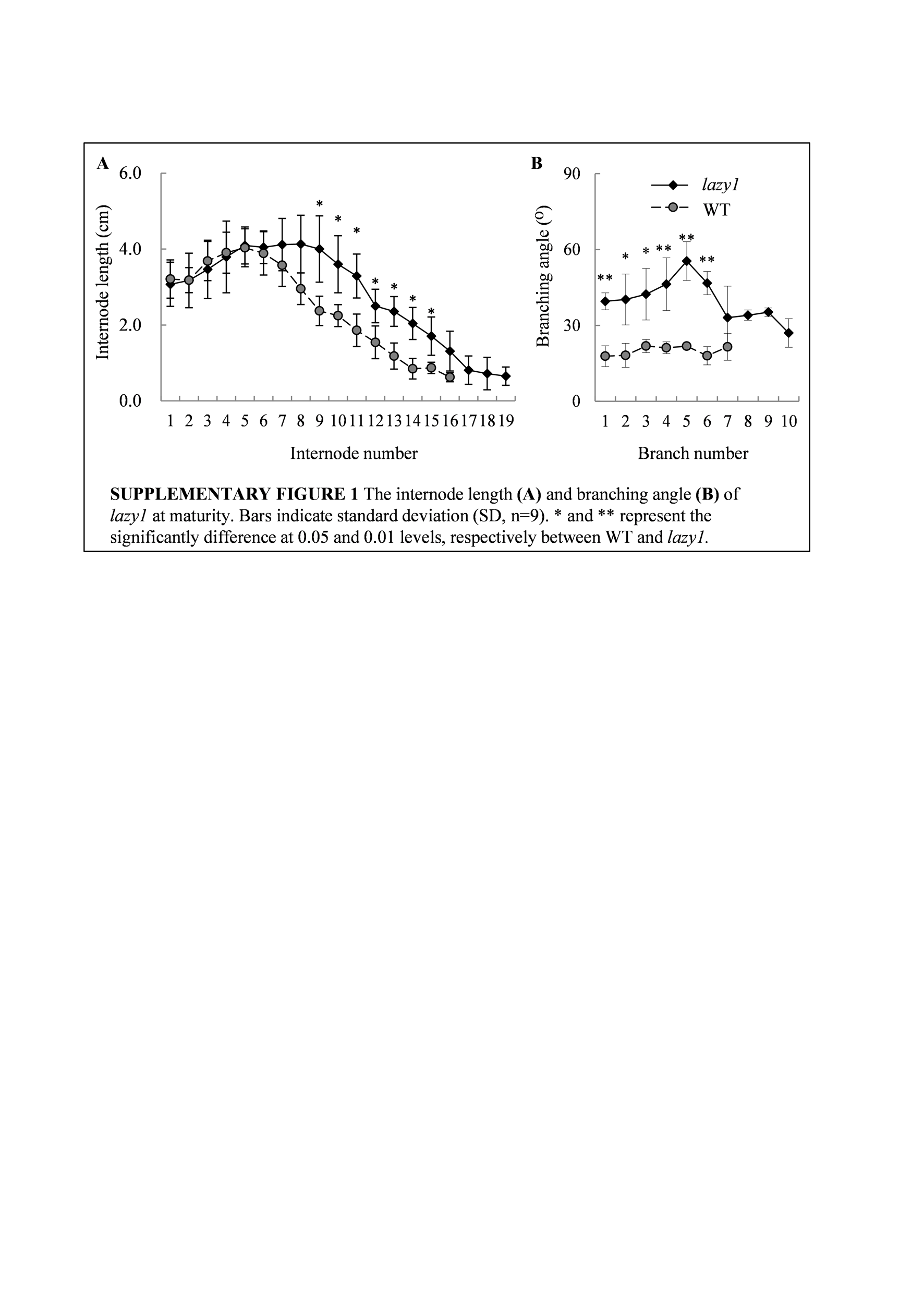

Supplement: Supplementary file 4 [file Image_1.TIF]

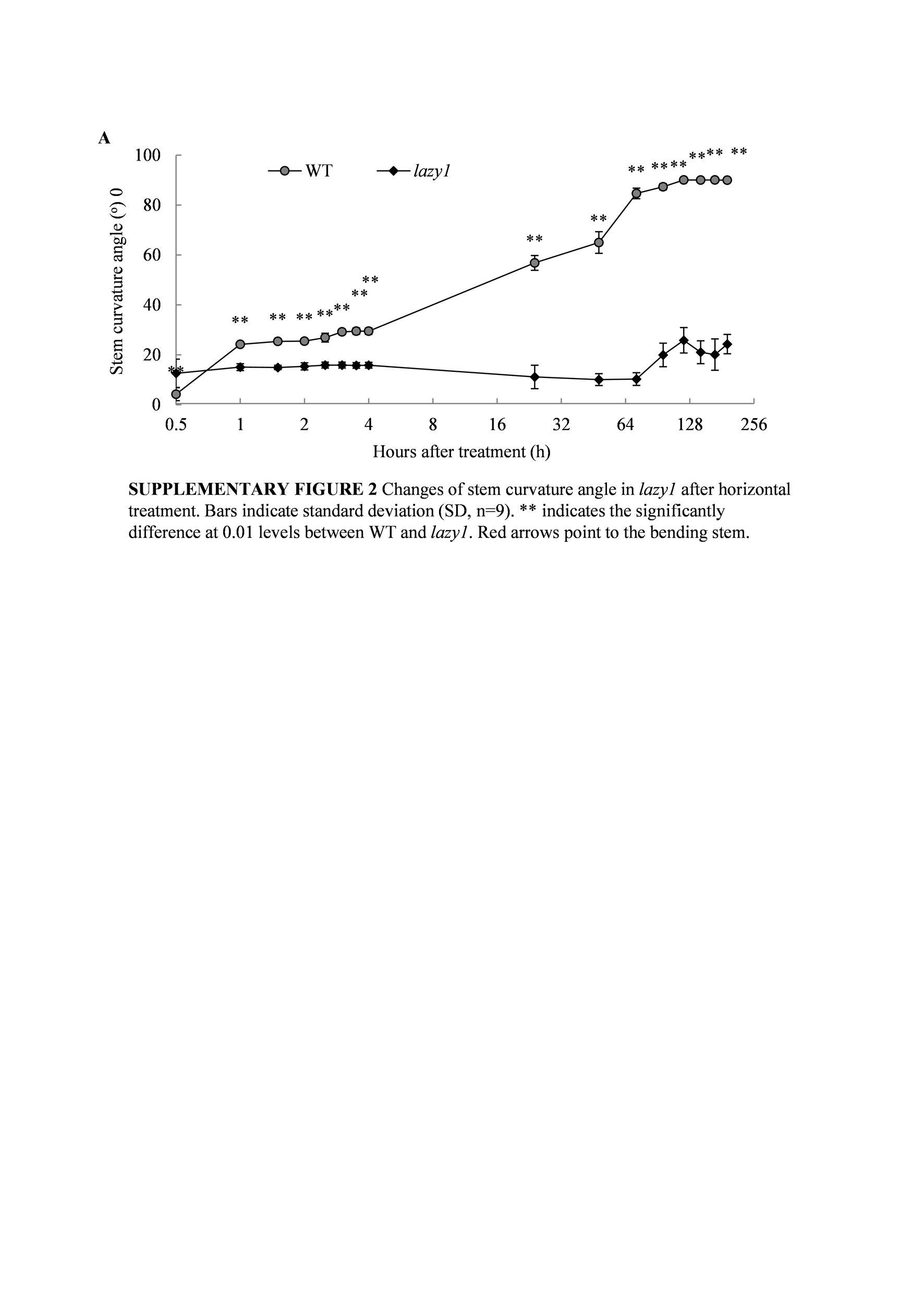

Supplement: Supplementary file 5 [file Image_2.TIF]

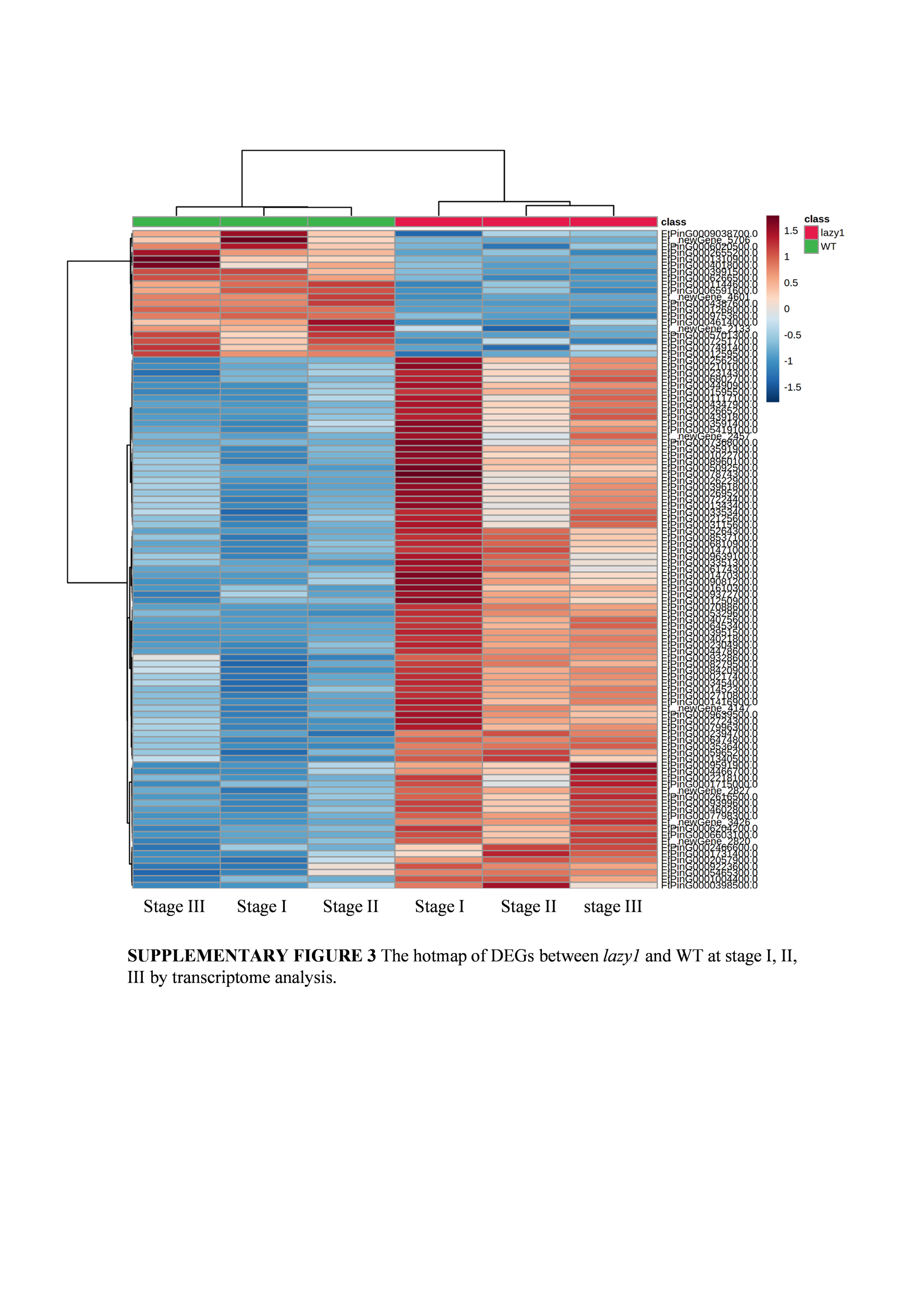

Supplement: Supplementary file 6 [file Image_3.TIF]

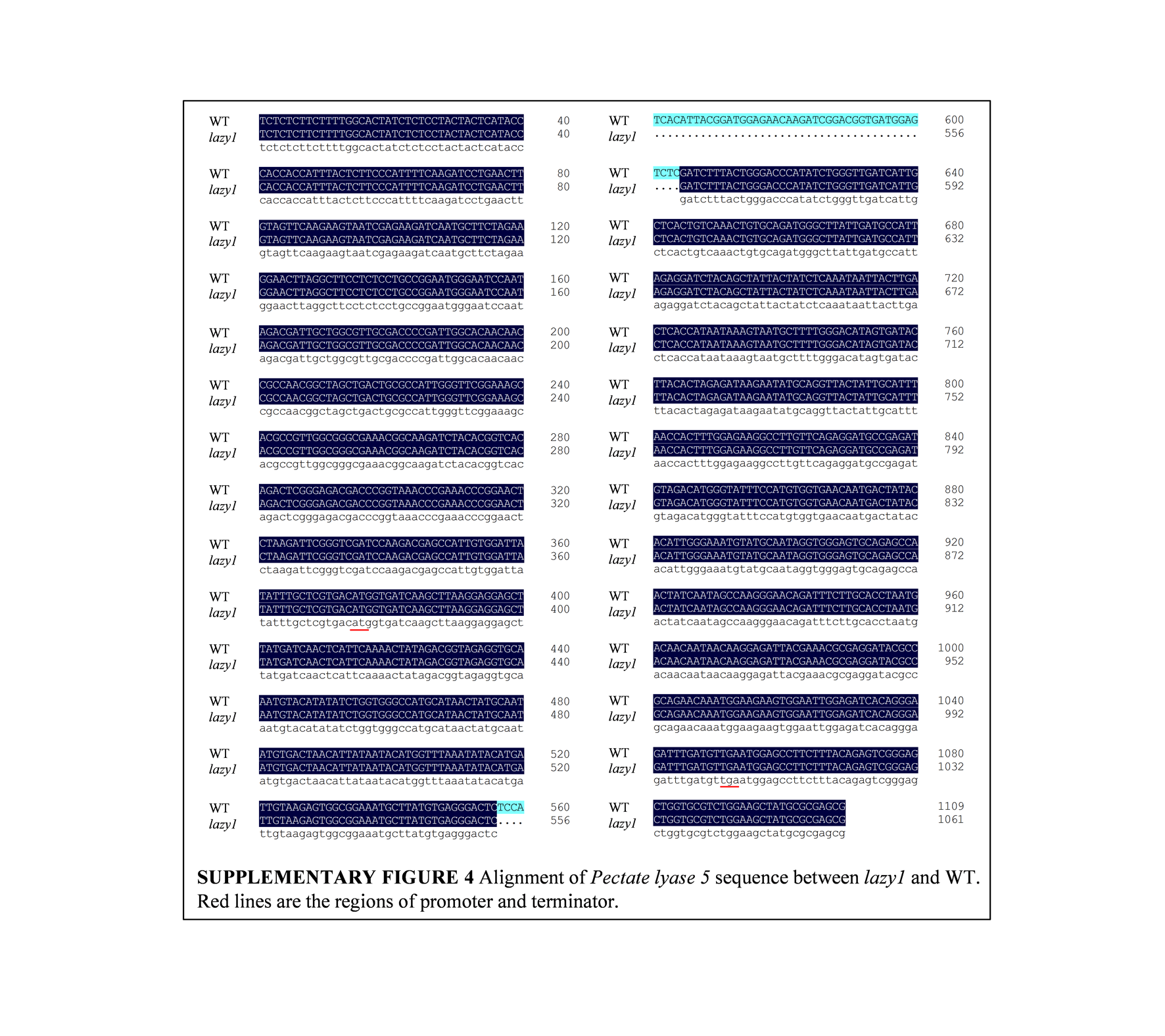

Supplement: Supplementary file 7 [file Image_4.TIF]

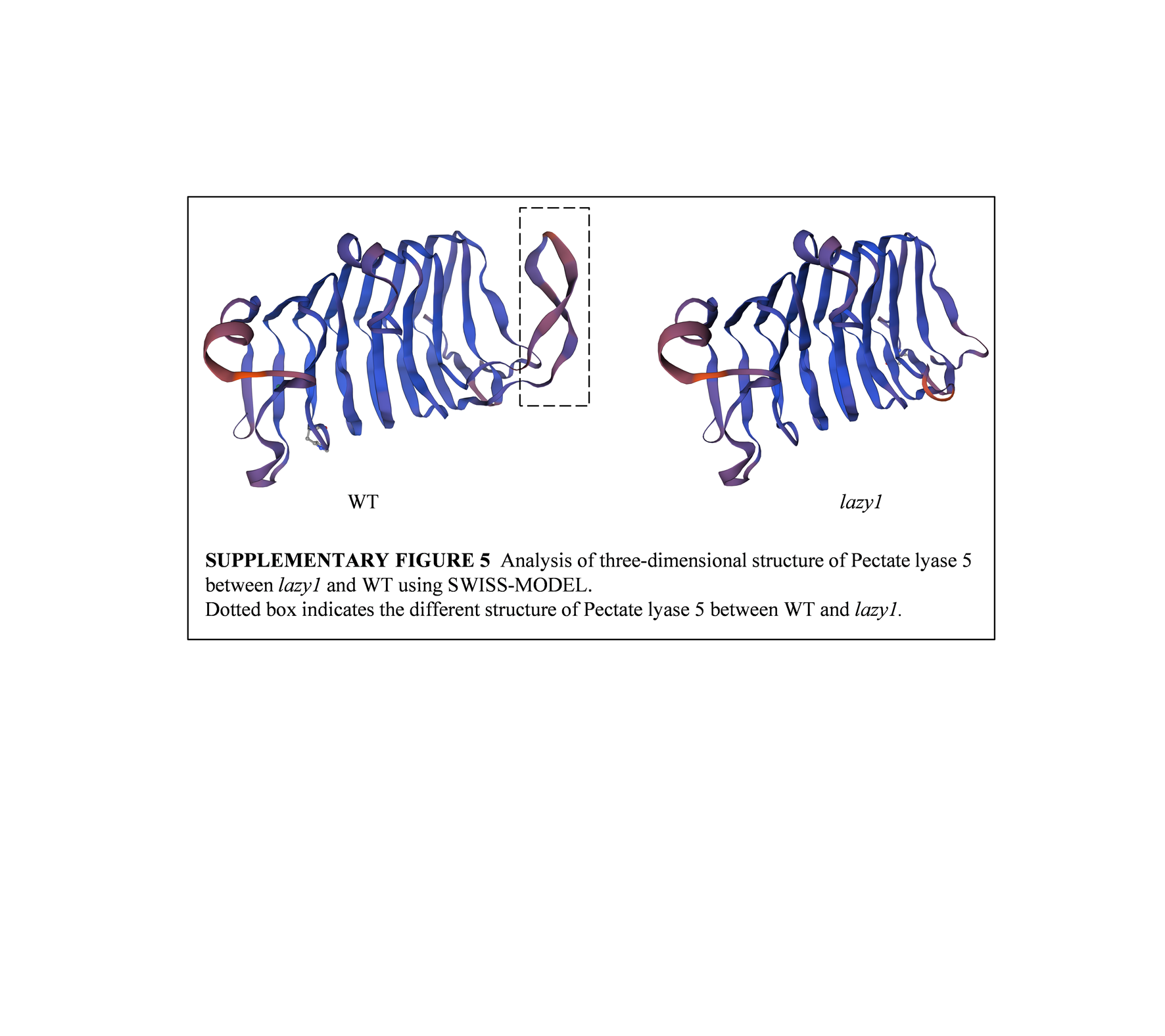

Supplement: Supplementary file 8 [file Image_5.TIF]
